# Chlordecone exposure and adverse effects in French West Indies populations

**DOI:** 10.1007/s11356-015-4621-5

**Published:** 2015-05-05

**Authors:** Luc Multigner, Philippe Kadhel, Florence Rouget, Pascal Blanchet, Sylvaine Cordier

**Affiliations:** Inserm, U1085 - IRSET, F-97145 Pointe-à-Pitre, Guadeloupe France; Université de Rennes 1, F-35700 Rennes, France; Service de Gynécologie-Obstétrique, CHU de Pointe à Pitre, F-97159 Pointe à Pitre, France; Service d’Urologie, CHU de Pointe à Pitre, F-97159 Pointe à Pitre, France; Université Antilles-Guyane, F-97159 Pointe-à-Pitre, France

**Keywords:** Cancer, Chlordecone, Development, Endocrine disruptor, Fertility, French West Indies, Insecticides, Organochlorine, Pregnancy

## Abstract

Chlordecone (Kepone) is an organochlorine insecticide that has been used as insecticide and fungicide. In the French West Indies, Guadeloupe and Martinique, it was intensively applied to banana fields from 1973 to 1993 to control root borers. This pesticide undergoes no significant biotic or abiotic degradation in the environment and is still present in soils where it was applied. It was only in 1999 that health and environmental authorities became aware of the extent of the chlordecone pollution of environmental media, including soils, waterways, and the food chain. Earlier observations and toxicological studies have demonstrated that chlordecone is a reproductive and developmental toxicant, neurotoxic and carcinogenic in rodents, and is an endocrine-disrupting chemical because of its estrogenic properties both in vitro and in vivo. Several surveys have confirmed that the French West Indian population continues to be exposed to this chemical though consumption of contaminated foodstuffs. Here, we report the findings of various epidemiological studies conducted in the French West Indies to assess the impact of environmental exposure to chlordecone on the health of the population.

## Introduction

Chlordecone is an organochlorine insecticide that was used intensively in the French West Indies, Guadeloupe and Martinique, from 1973 to 1993 to control banana root borers. As early as 1975, residual concentrations of this chemical were reported in soils previously used to grow bananas and subsequently converted to other agricultural activities, and in river water bordering banana fields (Snegaroff 1977). In 1980, a detailed report showed that chlordecone was the chlorinated chemical most frequently detected and at the highest concentrations in wild birds, mammals, fish, and shellfish living in areas of banana cultivation or at the coast near outlets of contaminated rivers (Cavelier 1980). Despite these warning signals, it was only from 1999 that French authorities and the population became aware of the extent of the pollution of environmental media. It was in 1999 that the implementation of the Drinking Water Directive of the European Union in the French West Indies revealed the presence of chlordecone at high concentrations (up to 10 μg/L) in numerous water resources. Later surveys conducted by environmental, health and food authorities and regulatory agencies revealed that it was extensively distributed in soils, rivers, spring and ground waters, aquatic biota, and crops (DIREN 2001; DSDS 2001; Dubuisson et al. 2007). These observations are not surprising because both the degradation of chlordecone whether physicochemical (abiotic) or microbial (biotic) is slow (Faroon et al. 1995). It has been estimated that the duration of chlordecone pollution of soil in French West Indies will last for decades or centuries (Cabidoche et al. 2009). Because a large proportion of tap water, local bottled spring water, and local foodstuffs were polluted and these products have been consumed, it was anticipated that human beings were also contaminated (Dubuisson et al. 2007). This was a major concern for health authorities and caused substantial anxiety in the population about the potential health implications.

## What we knew about chlordecone toxicity in 1999?

The toxicity and harmful effects of chlordecone exposure to humans was revealed in 1975 following a poisoning episode involving chlordecone plant workers in the industrial city of Hopewell, VA, USA (Cannon et al. 1978). Male workers showed evidence of prolonged toxicity involving the central nervous system (mainly appendicular intentional tremors, ataxia, oculomotor dysfunctions, slurred speech, irritability, mood disorders, and recent memory loss), enlarged liver and both low sperm cell counts and decreased percentages of motile sperm cells (Cannon et al. 1978; Cohn et al. 1978; Taylor 1982). These symptoms and clinical signs were grouped under the term of Kepone shake syndrome and were observed in subjects with chlordecone concentrations in blood over 1 mg/mL. The symptoms and clinical signs were reported to be reversible for most workers after cessation of exposure, and regression paralleled decreasing chlordecone concentrations in blood (Cohn et al. 1978; Taylor. 1982). The estimated blood half-life was estimated to be 120 to 160 days (Cohn et al. 1978). Detailed analysis of biological tissues and fluids revealed that the highest concentration of chlordecone was in the liver. Tissue-to-blood ratios (partition coefficient expressed for wet weight conditions) for the liver, fat, muscle, and gallbladder bile were 15, 6.7, 2.9, and 2.5, respectively (Cohn et al. 1978). Partition coefficients from liver or fat to blood (15 and 6.7, respectively) are lower than expected for a highly lipophilic organochlorine having a log *K*_*ow*_ of 5.41 (Hansch et al. 1995). This might be explained by the fact that chlordecone specifically binds to liver proteins and to plasma proteins, particularly albumin and high-density lipoproteins (HDL); this contrast with organochlorine pesticides which bind preferentially to VLDL and LDL lipoproteins and are preferentially distributed to fat tissues (Soine et al. 1982; Soine et al. 1984). Chlordecone (Kepone) is excreted in human bile as a reduced metabolite (chlordecone alcohol) and in the form of a glucuronide conjugate (Cohn et al. 1978). The bioreduction is catalyzed by a hepatic cytosolic enzyme termed chlordecone reductase (Molowa et al. 1986). However, only 5 % of the pesticide undergoing biliary excretion appears in stools each day, suggesting that it may be subject to an enterohepatic cycle process (intestinal reabsorption and recirculation to the liver) (Blanke et al. 1978).

Experimental studies with birds and mammals show effects similar to those reported in occupationally exposed humans including neurological effects, oligospermia, and hepatomegaly (reviewed in Faroon et al. 1995). Gestational and perinatal chlordecone exposures in rodents have been shown to be detrimental to normal fetal development and to impair neurobehavior during preweaning and postweaning development (Mactutus et al. 1982, 1984; Mactutus and Tilson 1984, 1985). Long-term feeding with chlordecone also produced an increased incidence of liver tumors mediated by tumor promotion rather than initiation (Reuber 1979; Sirica et al. 1989). Proteinuria, never observed in highly exposed men despite appropriate investigations (Guzelian 1982), was shown to be the most sensitive toxic endpoint in rats (Faroon et al. 1995) and is currently used as the basis for establishing acceptable levels of human exposure.

A great number of mechanisms have been suggested, but not always clearly demonstrated, to explain the toxicological profile of chlordecone. Nevertheless, hormone-like properties have been clearly established both in vivo and in vitro (Hammond et al. 1979; Eroschenko 1981): chlordecone has estrogenic properties that are now well established, and it is considered to be an endocrine-disrupting chemical.

## Health consequences of chlordecone exposure in the French West Indies

To date, there have been no publications, reports or testimonies of the presence of the Kepone shake syndrome in the French West Indies even among the banana farmers who used chlordecone. It is possible that their exposure was sufficiently low level for the internal bodily concentrations to have remained below the threshold (1 mg/L blood) for the onset of these severe clinical effects. However, we cannot completely rule out the possibility that there have been cases of this syndrome but that they were not recognized because of the diversity of signs and symptoms or that their relationship with chlordecone exposure was not recognized.

From 1999, various epidemiological studies have been initiated in the French West Indies to determine chlordecone exposure and its health consequences for the population.

### Male fertility

Chlordecone exposure affected sperm production and motility in men exposed at the Hopewell factory and experimental exposure has similar effects in rodents (Cohn et al. 1978; Linder et al. 1983).

In 1998, 1 year before the rediscovery of environmental pollution by chlordecone, a cross-sectional study was conducted to assess the fertility of banana workers and of men working in nonagricultural sectors in Guadeloupe. Blood and semen samples were collected from 100 adult men (Multigner et al. 2006; 2008). Further exploiting this study, the blood samples were later used to determine chlordecone body burden. Blood chlordecone was detected (limit of detection [LOD] ∼1 μg/L) in 88 % of men. The concentrations in banana workers (median 6.3 μg/L; range <LOD–104.5) were slightly but significantly higher than those in men who worked in nonagricultural sectors (median 5.5 μg/L; range <LOD–46.6). There were no significant differences between banana workers and workers in nonagricultural sectors for time to pregnancy for the last conception or for any semen characteristic (semen volume, sperm concentration, output, motility, and morphology) or hormone level (FSH, LH, Inhibin B, testosterone, and estradiol) (Multigner et al. 2008). Moreover, there were no significant correlations between chlordecone concentrations in blood and any semen characteristic or hormone concentration (Multigner et al. 2006). These results showed that current exposure levels, several years after the cessation of chlordecone use in banana fields, does not detectably affect male fertility; they thus agree with previous observations showing that the threshold for abnormally low seminal characteristics was of 1 mg/L (Cohn et al. 1978).

### Pregnancy complications and outcomes

Few women were affected by the chlordecone poisoning episode in Hopewell, and no studies could be done in pregnant women and newborns (Cannon et al. 1978). Studies on animals show that pregestational or gestational exposure of rats and mice affects implantation and prenatal and postnatal development (reviewed in Faroon et al. 1995).

In 2004, the blood chlordecone concentration was determined in 112 pregnant women at delivery in Guadeloupe (Hibiscus study) (Kadhel 2008): chlordecone was detected (LOD 0.50 μg/L) in 87 % (median 2.2 μg/L; range <LOD–16.5). Chlordecone was also assayed in subcutaneous abdominal fat from a sample (*n* = 52) of women who delivered by cesarean and detected in all samples (median 39.0 μg/kg lipids; range 25.1–207.1). The fat-to-blood ratio was 14 for chlordecone; the partition coefficient of other organochlorines in the same samples was 107 for p,p′-DDE and 362 for PCB153.

A prospective epidemiological mother–child cohort (Timoun study) was subsequently established in Guadeloupe to study the consequences of prenatal and postnatal chlordecone exposure on pregnancy and infant development. From 2004 to 2007, 1068 pregnant women were enrolled during the third-trimester checkup. At enrolment, the participants answered a standardized questionnaire covering sociodemographic characteristics, medical and obstetrical history, and lifestyle factors. After delivery, the medical history of the pregnancy and delivery information was collected. A semiquantitative food frequency questionnaire collected usual dietary intake during pregnancy. Maternal exposure in this cohort was attributed to the intake of contaminated food and water, especially seafood, root vegetables, and cucurbitaceous vegetables (Guldner et al. 2010). Maternal blood samples were obtained at delivery. Blood chlordecone was detected (LOD 0.06 μg/L) in 88 % of pregnant women (median 0.39 μg/L; range <LOD–19.3).

Hypertensive disorders and diabetes mellitus are common problems during pregnancy and are associated with significant short-term and long-term adverse health outcomes for both mothers and children. In a case–control study within the full Timoun cohort study, maternal chlordecone exposure was found to be associated with a significantly lower risk of gestational hypertension (odds ratio [OR] 0.2; 95 % confidence interval (CI) 0.1, 0.5, and OR 0.3; 95 % CI 0.2, 0.7, for the third and fourth quartiles of exposure, respectively) (Saunders et al. 2014). No significant associations were observed between chlordecone exposure and the risk of preeclampsia or gestational diabetes mellitus. The negative association observed between exposure to chlordecone and risk of gestational hypertension could be due to a hypotensive effect mediated by the sympathomimetic and/or progestin properties of the molecule (Hammond et al. 1979; Tilson et al. 1987).

The potential effects of maternal chlordecone exposure on length of gestation and preterm birth (birth before 37 completed weeks of gestation) have also been examined in the Timoun cohort study (Kadhel et al. 2014). The regression coefficient for length of gestation (β) was significantly lower for the fourth and fifth quintiles of exposure (−0.60, 95 % CI −0.99, −0.20, and −0.48, 95 % CI −0.88, −0.07, respectively). The hazard ratio (HR) for preterm birth was also significantly higher for the fourth and fifth quintiles of exposure (3.1, 95 % CI 1.6, 6.0, and 2.2, 95 % CI 1.1, 4.5, respectively). A one log_10_ increase in chlordecone concentration was associated with decreased length of gestation (β −0.26 week, 95 % CI −0.49, −0.03) and increased risk of preterm birth (HR 1.60; 95 % CI 1.1, 2.3). Parturition is triggered by the shortening and dilatation of the cervix associated with uterine contractions. Progesterone plays a key role in maintaining pregnancy, and treatment of pregnant women with progesterone receptor antagonists induces labor at any stage of pregnancy (Chwalisz and Garfield 1994). Chlordecone binds estrogen receptors (ER) and stimulates the synthesis in vivo of the progesterone receptor in rat uterine tissues (Hammond et al. 1979), and this process is mediated by ER. These observations suggest that the observed associations between exposure to chlordecone and decreased gestational length and increased risk of preterm birth are due to estrogenic and/or progestin activities of chlordecone.

### Early child development

Chlordecone crosses the placental barrier in pregnant rodents and transfers to the newborn through maternal breastfeeding, thus exposing the developing organism during the earliest stages of development (Kavlock et al. 1980).

In the Hibiscus study, the blood cord of 112 newborns and colostrum of mothers who intended to breastfeed were assayed for chlordecone (Kadhel 2008). Chlordecone was detected (LOD 0.50 μg/L) in 61 % of cord blood samples (median 0.7; range <LOD–3.7) and 39 % of colostrum samples (range of detectable values 0.5–2.8 μg/L). The milk-to-blood ratio was 1/10 for chlordecone; the partition coefficient of other organochlorines in the same samples was 6 for p,p′-DDE and 7 for PCB153. The partitioning of chlordecone is the consequence of specific transporters in blood (see above). Indeed, chlordecone is transported by HDL lipoproteins which promote transport to the liver and not to peripheral fat or breast milk, the latter being free of triglycerides and type HDL cholesterol.

In the Timoun cohort study, cord blood samples were obtained at delivery and breast milk samples were collected 3 months after delivery. Chlordecone was detected (LOD 0.06 μg/L) in 56 % of the cord blood samples (median 0.25 μg/L; range <LOD–22.9) and 77 % of the breast milk samples (median 0.10 μg/L; range <LOD–0.34). In a follow-up of this cohort, infants were examined at 7 months of age to evaluate their visual acuity, cognition, and motor development using the Teller visual acuity card test II, the Fagan test of infant intelligence, and the Brunet-Lezine scale of psychomotor development (Dallaire et al. 2012). At 18 months of age, infant development was assessed with the ages and stages questionnaire (ASQ), a parent-completed screening test aimed at identifying children at risk of developmental delay (Boucher et al. 2013). Maternal interviews were conducted at these times to assess both potential confounders pertaining to sociodemographic and psychosocial domains, and the quality of stimulation provided by the family.

At 7 months, a dose-dependent decrease in novelty preference with higher cord chlordecone concentrations was observed (β −0.19, 95 % CI −0.35, −0.03, for the third tertile of exposure) (Dallaire et al. 2012). The preference for novelty tasks (longer fixations on new stimuli than on previously presented stimuli) is an indicator of short-term visual memory in infants (Fagan and Singer 1983). This result is consistent with reports of poorer short-term memory among workers in Hopewell (Cannon et al. 1978). In rodent models, preweaning rats exposed to chlordecone during the neonatal period exhibited memory deficits, particularly in early learning and information retention in passive avoidance tests (Mactutus et al. 1982; Mactutus and Tilson 1984).

Detectable levels of chlordecone in cord blood were also associated with higher risk of low scores on the fine motor development scale at 7 months (OR 1.26, 95 % CI 1.09, 1.47) (Dallaire et al. 2012). At 18 months of age, high chlordecone concentrations (as continuous value) in cord blood were still associated with poorer fine motor scores (β −0.24, *p* 0.03) (Boucher et al. 2013). This is in accordance with the observation of intention tremors in the exposed Hopewell employees and fine body tremors in rats after neonatal exposure to chlordecone (Cannon et al. 1978; Mactutus et al. 1984). At 18 months, analyses were also conducted separately for boys and girls, and this effect was only observed among boys (β −0.32, *p* 0.03), possibly related to chlordecone’s estrogen-like activity.

Recently, the impact of perinatal exposure to chlordecone on the thyroid hormone system at 3 months of age and on subsequent neurodevelopment was explored (Cordier et al. 2015). Cord chlordecone was associated with an increase in TSH in boys, whereas postnatal exposure (breastfeeding) was associated with a decrease in FT3 overall, and in FT4 in girls. Higher TSH concentrations at 3 months were positively associated with the ASQ score for fine motor development at 18 months, but TSH did not modify the association between prenatal chlordecone exposure and poorer ASQ fine motor score for boys. This suggests that the developmental toxicity of chlordecone may be mediated by the estrogen signaling pathway rather than by, or in addition to, the thyroid endocrine system

### Prostate cancer

Chlordecone is a potential carcinogen and causes hepatic tumors in laboratory rats and mice (Reuber 1979). A few hundred men were affected by the poisoning episode in Hopewell, a number insufficient for the long-term consequences on the incidence rare conditions, such as cancers, to be studied. The carcinogenic and hormonal properties of chlordecone and its long biological half-life make long-term effects, such as cancer, entirely plausible. In the particular context of the French West Indies, it was assumed that populations were first exposed to chlordecone in the early 1970s. Because chlordecone is a recognized endocrine-disrupting chemical, the potential relationship with hormone-dependent cancer was considered. A population-based case–control study was carried out in Guadeloupe from 2005 to 2007 to investigate the relationship between exposure to chlordecone and the risk of prostate cancer, the most common cancer in this population (Mallick et al. 2005). A total of 623 men with newly diagnosed prostate cancer and 671 controls were compared (Multigner et al. 2010). Exposure was determined from current plasma concentration: chlordecone was detected (LOD 0.25 μg/L) in 67 % of the subjects (median 0.6 μg/L; range <LOD–49.1). A significant positive association was found between the chlordecone concentration in blood and prostate cancer (OR 1.77; 95 % CI 1.21, 2.58, for the highest tertile of values above the LOD). These data were recently reanalyzed using, after validation of quality control charts, a LOD of 0.06 μg/L, and handling missing data by multiple imputation (Emeville et al. 2015). The significant positive association between chlordecone exposure and prostate cancer risk was confirmed (OR 1.65, 95 % CI 1.09, 2.48, for the highest quintile of exposure). Comparable results were observed when p,p′-DDE or PCB153 concentrations were included in the full model (OR 1.64, 95 % CI 1.09, 2.47, and OR 1.70, 95 % CI 1.12, 2.56, respectively).

Chlordecone may act as a tumor promoter, through hormone-mediated effects. Chlordecone binds the ER α (ERα) and β (ERβ), acting as an agonist of ERα and an antagonist of ERβ (Kuiper et al. 1998; Lemaire et al. 2006). ERα mediates the adverse effects of estrogen on the prostate, including aberrant proliferation, inflammation, and malignancy. ERβ exerts opposite and beneficial effects, such as antiproliferative, anti-inflammatory, and, potentially, anticarcinogenic effects (Ellem and Risbridger 2009). The interplay between the agonistic effects of chlordecone on ERα and its antagonistic effects on ERβ may increase proliferation of estrogen-sensitive tissues, increasing the risk of cancer.

## Conclusions

Chlordecone (Kepone) is now one of the environmental chemical agents most extensively studied for its effects on humans. More than 5 years of clinical investigations of workers heavily exposed in US and 10 years of epidemiological studies in the general population of the French West Indies exposed at environmental levels have established the spectrum of human toxicity of chlordecone. From 1999 to date, measurement of chlordecone in blood samples has revealed that a large proportion of the French West Indies population is still contaminated. Although exposure levels do not seem to have reached those that trigger the onset of the Kepone shake syndrome, epidemiological studies have revealed that there are effects during critical windows of exposure (pregnancy and infant development) and possibly long-term effects such as cancer. Further efforts are required to protect the population against exposure to chlordecone, particularly by reducing its intake through the diet.
